# Biomechanical and Clinical Study of Rod Curvature in Single-Segment Posterior Lumbar Interbody Fusion

**DOI:** 10.3389/fbioe.2022.824688

**Published:** 2022-03-03

**Authors:** Lin Han, Yongheng Li, Zhiyong Li, Hongdao Ma, Chenfeng Wang, Qiang Chen, Xuhua Lu

**Affiliations:** ^1^ Department of Orthopaedics, Shanghai Changzheng Hospital, Second Military Medical University, Shanghai, China; ^2^ Biomechanics Laboratory, School of Biological Science and Medical Engineering, Southeast University, Nanjing, China; ^3^ School of Mechanical Medical and Process Engineering, Queensland University of Technology, Brisbane, QLD, Australia

**Keywords:** rod curvature, rod contouring, lumbar surgery, finite element analysis, PLIF surgery

## Abstract

**Objective:** Pedicle screw fixation is a common technique used in posterior lumbar interbody fusion (PLIF) surgery for lumbar disorders. During operation, rod contouring is often subjective and not satisfactory, but only few studies focused on the rod-contouring issue previously. The aim of the study was to explore the effect of the rod contouring on the single-segment PLIF by the finite element (FE) method and retrospective study.

**Methods:** A FE model of the lumbosacral vertebrae was first reconstructed, and subsequently single-segmental (L4/5) PLIF surgeries with four rod curvatures (RCs) were simulated. Herein, three RCs were designed by referring to centroid, Cobb, and posterior tangent methods applied in the lumbar lordosis measurement, and zero RC indicating straight rods was included as well. Clinical data of patients subjected to L4/5 segmental PLIF were also analyzed to verify the correlation between RCs and clinical outcome.

**Results:** No difference was observed among the four RC models in the range of motion (ROM), intersegmental rotation angle (IRA), and intradiscal pressure (IDP) under four actions. The posterior tangent model had less maximum stress in fixation (MSF) in flexion, extension, and axial rotation than the other RC models. Patients with favorable prognosis had larger RC and positive RC minus posterior tangent angle (RC-PTA) of fused segments with respect to those who had poor prognosis and received revision surgery.

**Conclusion:** All RC models had similar biomechanical behaviors under four actions. The posterior tangent-based RC model was superior in fixation stress distribution compared to centroid, Cobb, and straight models. The retrospective study demonstrated that moderate RC and positive RC-PTA were associated with better postoperative results.

## Introduction

Optimal sagittal alignment plays a significant role in improving the spinal sagittal balance, reducing body energy expenditure, and slowing down the disc degeneration of adjacent segments (Makhni et al., 2018). Loss of the spinal sagittal balance can be caused by disc degeneration, spinal deformity, trauma, and surgery, and thus higher muscular force is required to maintain the spinal posture and balance. However, the compensatory mechanism may result in adverse effects such as back pain, disability, and decrease in health-related quality of life (HRQOL). In addition, positive sagittal balance indicated by abnormal radiographic parameters is also highly correlated to the adverse outcomes in adult spinal deformity (Glassman et al., 2005).

Restoration of normal sagittal alignment is critical to postoperative outcomes and prognosis (Matsumoto et al., 2017). After spinal fusion, increased stress on the adjacent discs may result in adjacent segment degeneration (ASD). It was reported that the incidences of radiograph ASD and symptomatic ASD were 26.6 and 8.5% in lumbar surgery, respectively (Xia et al., 2013). Sagittal imbalance, such as sagittal vertical axis (SVA) > 50 mm, higher pelvic tilt (PT), decreased lumbar lordosis (LL), and pelvic incidence minus lumbar lordosis (PI-LL) mismatch, is identified as a significant risk factor of ASD after posterior lumbar interbody fusion (PLIF) (Barrey and Darnis, 2015). This pathological change can cause neurologic symptoms which require further medical interventions.

In view of the significance of the normal sagittal alignment, the posterior screw–rod system as the main fixation device and rod curvature (RC) should be consistent with the physiological alignment to achieve satisfactory postoperative outcomes. Otherwise, negative postoperative outcomes can be introduced. For instance, changes in the bending curvature of the implanted rod resulted in overcorrection or undercorrection of the sagittal balance in adolescent idiopathic scoliosis (Salmingo et al., 2014), and the mismatch between RC and normal sagittal alignment correlated to the poor clinical and radiological follow-up (Moufid et al., 2019).

However, perioperative rod contouring or rod bending was rarely studied previously. In clinical practice, French bender is the most commonly used tool for rod contouring. The surgeon uses the device to bend rods according to experience and preference after evaluating the sagittal alignment of patients from the radiography during the operation. Obviously, this experience- or preference-based practice is inevitably subjective and poorly repeatable. Moreover, repetitive rod contouring is likely to cause imprecise fixation, screw loosening, stress concentration, and long operation duration. Therefore, evaluating the rod contouring indicated by RC is necessary. Herein, finite element (FE) analysis was adopted to evaluate the biomechanical effects of four RCs on the single-segment PLIF surgery including three kinds of contoured rods corresponding to three commonly used LL measuring methods (*i.e.*, centroid, Cobb, and posterior tangent) and straight rod. Then, a retrospective clinical study was conducted to discuss whether the RC was correlated with the clinical outcome.

## Materials and Methods

### Finite Element Modeling

**FIGURE 1:**
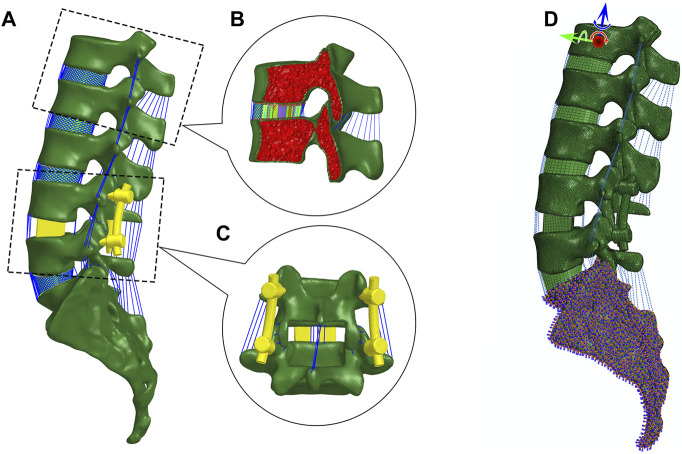
Finite element modeling. **(A)** Lateral view of the lumbosacral model with posterior lumbar interbody fusion in Hypermesh. **(B)** Sagittal section of vertebrae and intervertebral disc. The vertebra is partitioned into cortical and cancellous bone. The disc is composed of nucleus pulposus and annulus ground substance embedded with crossing collagen fibers. **(C)** Posterior view of PLIF. **(D)** Boundary and loading conditions.

A three-dimensional FE model of the lumbosacral vertebrae (Figure 1) was first reconstructed through Mimics 16.0 software (Materialise, Leuven, Belgium), based on computed tomography images (CT, Philips Brilliance iCT 256; slice thickness, 1 mm; scanning voxel size, 0.8 × 0.8 × 1.2 mm^3^) of a normal male adult without any lumbar disease. The participant was provided a written informed consent prior to the enrollment, and the study protocol was approved by the Institutional Ethics Committee.

Regarding the FE model, the cortical bone, cancellous bone, endplates, and intervertebral disc were created in 3-matic 8.0 (Materialise, Leuven, Belgium) and then meshed in HyperWorks 13.0 (Altair Engineering, Inc., Executive Park, CA, United States). Cortical bone was separated from each vertebra with a thickness of 1 mm (Naserkhaki et al., 2018), and the rest of the vertebra was treated as cancellous bone. The thickness of the endplate was set at 0.5 mm. The element types of vertebrae and endplates were four-node tetrahedral element (C3D4) and eight-node hybrid hexahedral elements (C3D8H), respectively. Intervertebral discs were divided into incompressible nucleus pulposus and annulus ground substance (Guo and Fan, 2018). The nucleus pulposus and annular ground substance were meshed by the C3D8H element with an isotropic, hyper-elastic Mooney–Rivlin material. Six circumferential layers of crossing collagen fibers with different orientations were embedded in the annulus ground substance, and the fibers were meshed by tension-only two-node truss elements (T3D2). A total of seven major ligaments including anterior longitudinal ligament (ALL), posterior longitudinal ligament (PLL), ligamentum flavum (LF), capsular ligament (CL), intertransverse ligament (ITL), interspinous ligament (ISL), and supraspinous ligament (SSL) were meshed by tension-only Spring A elements (Rohlmann et al., 2006). A frictionless surface contact between facet joints was assigned. All material properties of the aforementioned tissues were listed in Table 1.

**TABLE 1:** Material properties of the FE model.

Component	Element type	Young’s modulus (Mpa)		Poisson’s ratio		Density (g/cm^3^)		Cross section (mm^2^)		Reference
Bone										
Cortical bone	C3D4	12,000		0.3		1.7e-6				Goel et al. (1994)
Cancellous bone	C3D4	100		0.2		1.1e-6				
Endplate	C3D8H	23.8		0.4		1.2e-6				Ueno and Liu (1987)
**Intervertebral disc**										
Annulus ground substance	C3D8H	C_10_ = 0.18, C_01_ = 0.045				1.05e-6				Schmidt et al. (2007)
Nucleus pulpous	C3D8H	C_10_ = 0.12, C_01_ = 0.03				1.02e-6				
Annulus fiber layers										Polikeit et al. (2003)
Outermost	T3D2	550		0.3		1.0e-6		0.70		
Second	T3D2	495		0.3		1.0e-6		0.63		
Third	T3D2	440		0.3		1.0e-6		0.55		
Fourth	T3D2	420		0.3		1.0e-6		0.49		
Fifth	T3D2	385		0.3		1.0e-6		0.41		
Innermost	T3D2	360		0.3		1.0e-6		0.30		
**Fixation devices**										
Screw and rod (Ti6Al4V)	C3D4	113,000		0.3						
Cage (PEEK)	C3D8H	3,500		0.3						
** Ligaments**	**Element type**	**Strain (%)**	**Stiffness (N/mm)**	**Strain (%)**	**Stiffness (N/mm)**	**Strain (%)**	**Stiffness (N/mm)**	**Strain (%)**	**Stiffness (N/mm)**	
Anterior longitudinal ligament	Spring A	ε < 0	0	0< *ε* < 12.2	347	12.2< *ε* < 20.3	787	20.3 < *ε*	1864	Rohlmann et al. (2006)
Posterior longitudinal ligament	Spring A			0< *ε* < 11.1	29.5	11.1< *ε* < 23	61.7	23 < *ε*	236	
Ligamentum flavum	Spring A			0< *ε* < 5.9	7.7	5.9< *ε* < 49	9.6	49 < *ε*	58.2	
Intertransverse ligament	Spring A			0< *ε* < 18.2	0.3	18.2< *ε* < 23.3	1.8	23.3 < *ε*	10.7	
Capsular ligament	Spring A			0< *ε* < 25	36	25< *ε* < 30	159	30 < *ε*	384	
Interspinous ligament	Spring A			0< *ε* < 13.9	1.4	13.9< *ε* < 20	1.5	20 < *ε*	14.7	
Supraspinous ligament	Spring A			0< *ε* < 20	2.5	20< *ε* < 25	5.3	25 < *ε*	34	

### Contouring Methods and Simulation of the Posterior Lumbar Interbody Fusion Procedure

**FIGURE 2:**
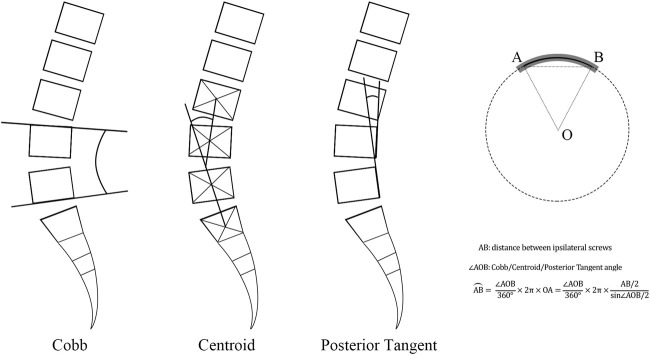
Description of Cobb, centroid, and posterior tangent methods in the rod bending procedure.

Centroid, Cobb, and posterior tangent methods (Figure 2) were applied to measure angles of L4–L5 segment curvatures in Mimics (Harrison et al., 2001). Here, the centroid angle was described as the included angle made by two straight lines, which passed through two vertebral centroids at both ends (Chen, 1999). The Cobb angle was defined as the angle between the superior endplate of L4 and the inferior endplate of L5. The posterior tangent angle was defined as the included angle of two lines passing tangentially to posterior wall of the L4 and L5 end vertebrae (Janik et al., 1998). The shape of the contoured rod was simply treated as an arc of a virtual circle. According to the distance between ipsilateral screw tails (*i.e.*, chord length of the arc) and the three previously defined angles, the arc length (*i.e.*, the rod contour) was calculated. The RCs of centroid, Cobb, and posterior tangent models were 33.25°, 18.65°, and 7.99°, respectively. In addition, the straight model (RC = 0°) was also considered. Accordingly, the four types of rods were modeled in SolidWorks 2003 (SolidWorks Corp., Waltham, MA, United States).

PLIF was modeled on L4–L5 segment in 3-matic software. According to the surgical procedure, pedicle screws with a diameter of 6 mm were inserted along the central axis of the pedicle, and two screws in L4 and L5 were parallel to their superior endplates, respectively. Partial spinous processes and laminae (inferior L4 and superior L5) and half of the medial facet joints and three ligaments (LF, ISL, and SSL between L4 and L5) were removed. Then, two cubic cages were inserted into the disc space after removing the inferior endplate of L4 and superior endplate of L5, and this simulated the clinical practice. Finally, rods with four RCs were assembled in each model. All fixation devices were meshed in HyperWorks. Tie constraints were employed to model the contact between vertebrae and screws or cages. All material properties of fixation devices were also reported in Table 1.

### Boundary and Loading Conditions

The six degrees-of-freedom of the sacrum were constrained. The loading history involved two steps. In the first step, a preload of 500 N as a follower load representing upper body weight and muscle forces was applied according to the force line of the lumber spine (Patwardhan et al., 1999). In the second step, a 7.5 N m moment was applied on the top center of the L1 vertebral body in four actions, *i.e.*, flexion, extension, lateral bending, and axial rotation (Ayturk and Puttlitz, 2011). FE simulation was performed by Abaqus (v6.14, Simulia Inc., Providence, RI, United States).

### Model Validation and FE Analysis

To validate the FE model, a normal lumbosacral model was also developed (Supplementary Figure S1 in Supplementary Materials), and the range of motion (ROM) of the normal lumbosacral model and L4/5 intradiscal pressure (IDP) were compared with a recent FE study and an *in vitro* biomechanical experiment (Rohlmann et al., 2001; Wang et al., 2018). To be consistent with the literature, a follower load of 280 N and increasing moments from 0.0 to 7.5 N m with an interval 2.5 N m were applied on the FE model in flexion, extension, lateral bending, and axial rotation tests. After the validation, ROM, intersegmental rotation angle (IRA) of adjacent levels, IDP in adjacent segmental discs, and maximum von Mises stress in fixation (MSF) of the four RC models were compared (Huang et al., 2021).

### Clinical Study

**FIGURE 3:**
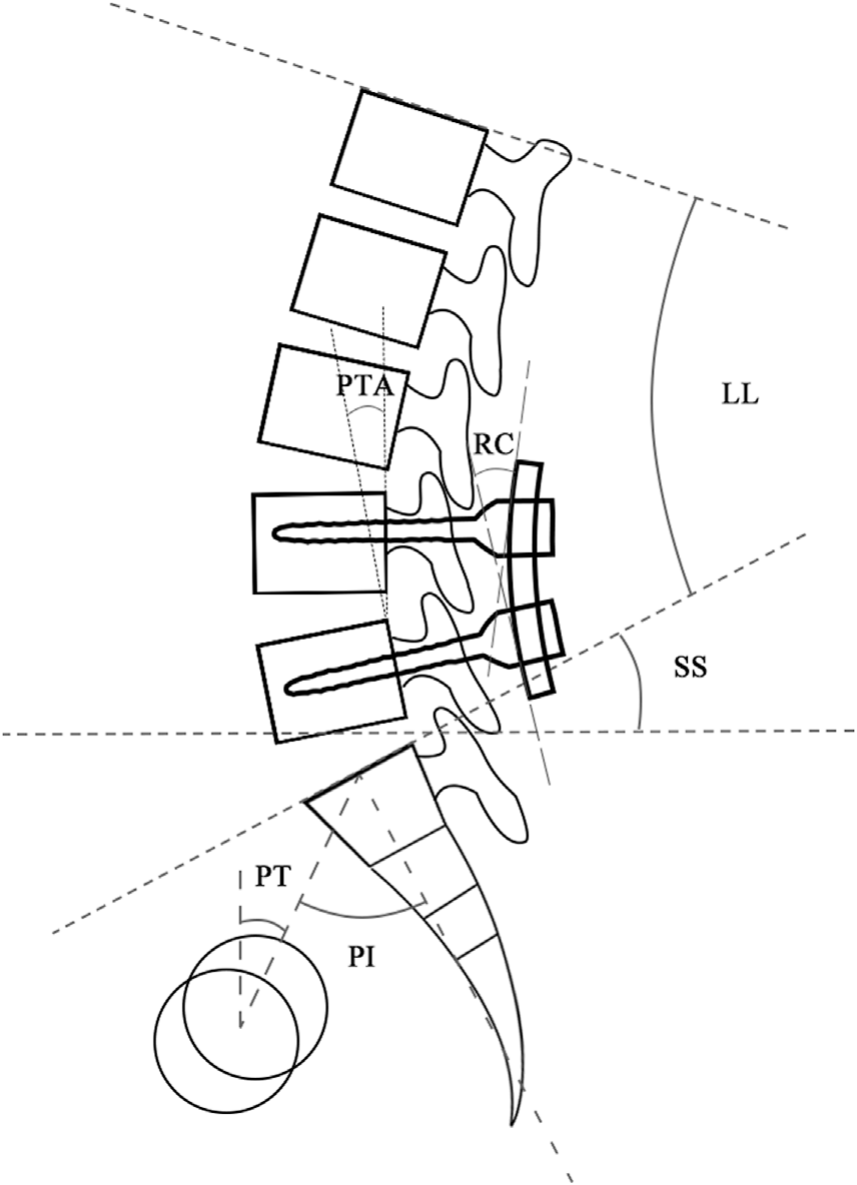
Radiographic parameters measured from lateral radiographs. Abbreviation: LL, lumbar lordosis; PT, pelvic tilt; PI, pelvic incidence; SS, sacral slope; RC, rod curvature; PTA, posterior tangent angle of fused segments.

The retrospective study identified patients subjected to L4/5 segmental PLIF surgery for lumbar spinal stenosis from January 2015 to June 2021 with the Institutional Review Board’s (IRB) approval. The exclusion criteria were patients with the diagnosis of spinal infection, injury, tumor, apparent deformity, irreducible lumbar spondylolisthesis, and other diseases that caused spinal instability. The normal group included patients with an over two-year satisfactory follow-up, whereas the abnormal group consisted of patients who had poor recovery within two-year follow-up after primary surgery and received revision surgery. Patient demographics (age, gender, and BMI), surgical time, and hospital stay were collected. Radiographic parameters including LL, PT, PI, sacral slope (SS), PI-LL, RC, posterior tangent angle of fused segments (PTA), and the difference between RC and PTA (RC-PTA) were measured from postoperative lateral radiographs (Figure 3). The χ^2^ test for the parameter of gender and student *t*-test for the remaining parameters were used to determine statistical difference between groups (SPSS Statistics 16.0, IBM Corporation, Somers, NY, United States), and *p* < 0.05 indicated the statistical significance.

## Results

### Model Validation

**FIGURE 4:**
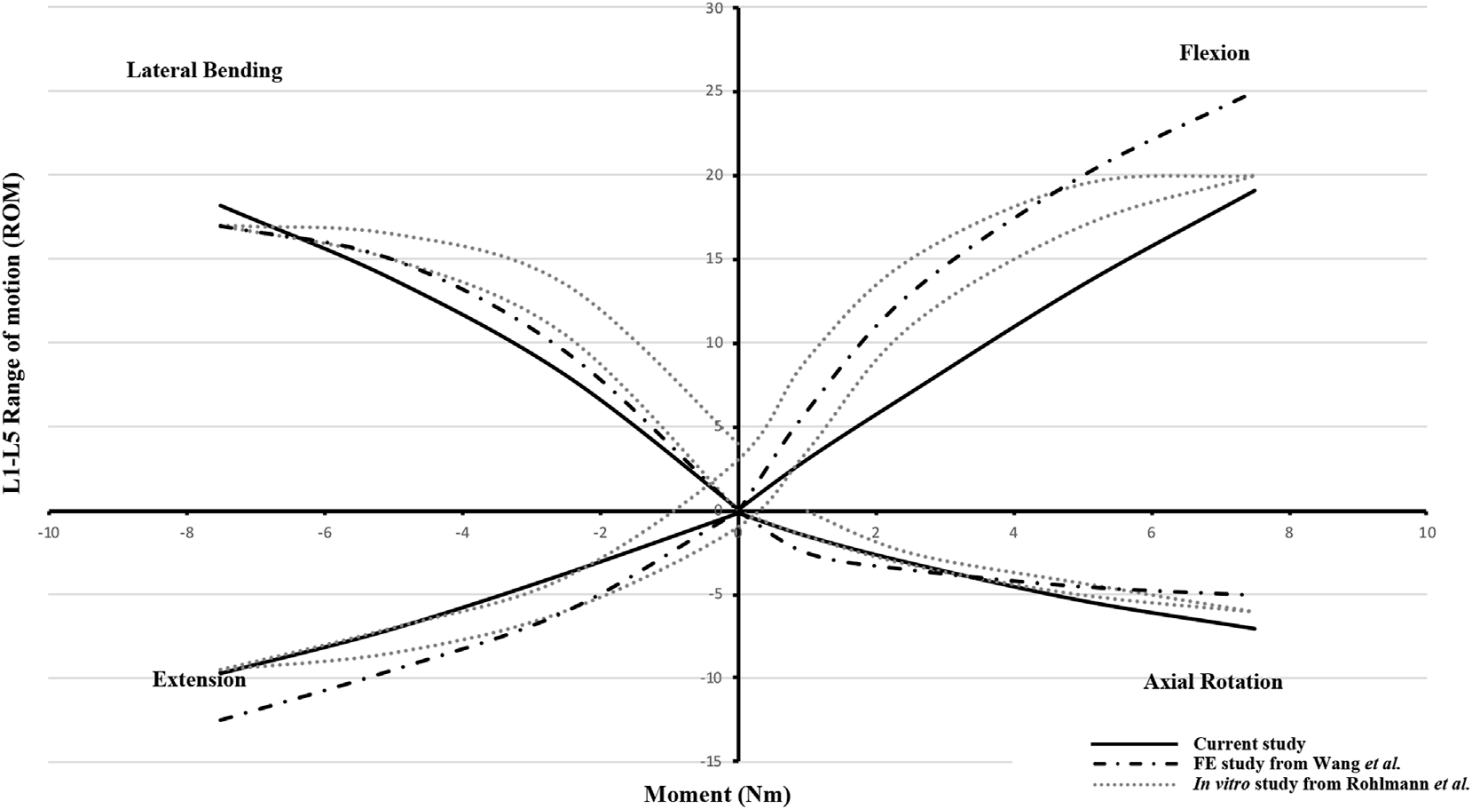
Range of motion (ROM)–moment curvatures for model validation versus the FE study from Wang *et al.* and the *in vitro* study from Rohlmann *et al.*

**FIGURE 5:**
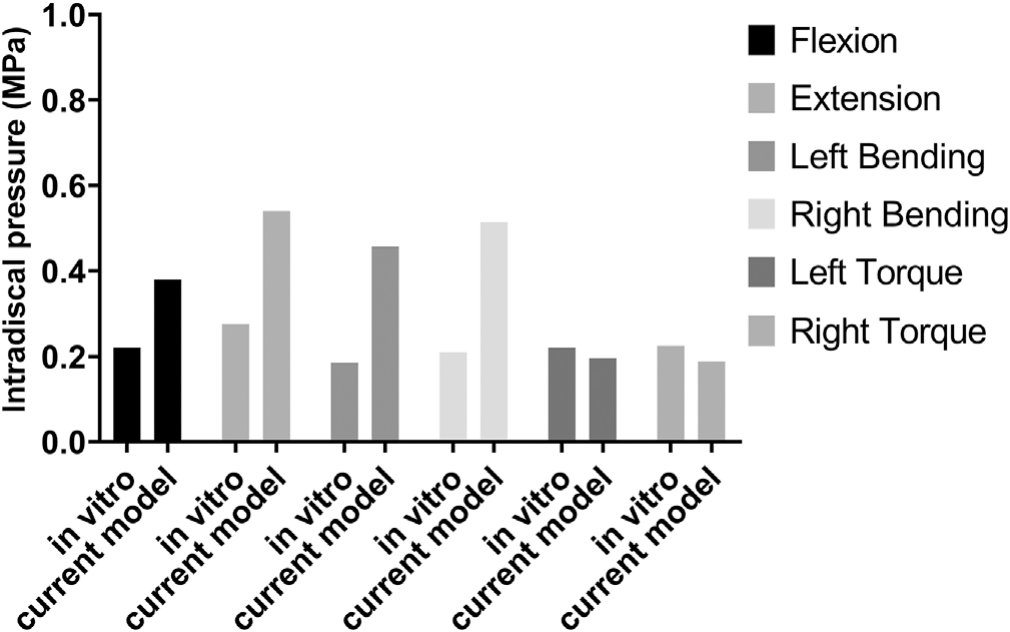
Intradiscal pressure (IDP) of the L4/5 disc for model validation by comparing the *in vitro* study and current model.

The ROM of the normal lumbosacral model was validated against two previous studies (Rohlmann et al., 2001; Wang et al., 2018). As shown in Figure 4, the ROM–moment curves in the current study agree with the ranges of the *in vitro* study and FE study. The distribution of the IDP in L4/5 is similar to that of the *in vitro* study, *i.e.*, extension > right bending > left bending > flexion > left torque > right torque. However, compared to the axial torsion, the IDPs of flexion, extension, and lateral bending between the present FE result and the *in vitro* experiment show a discrepancy (Figure 5). The reason might be that residual muscles or tendons connecting the spine in the *in vitro* study reduced the stress level of the IDP. In any case, IDPs in the current study are still in the acceptable ranges (Dreischarf et al., 2014; Naserkhaki et al., 2018).

### Biomechanical Analysis of the Groups

**FIGURE 6:**
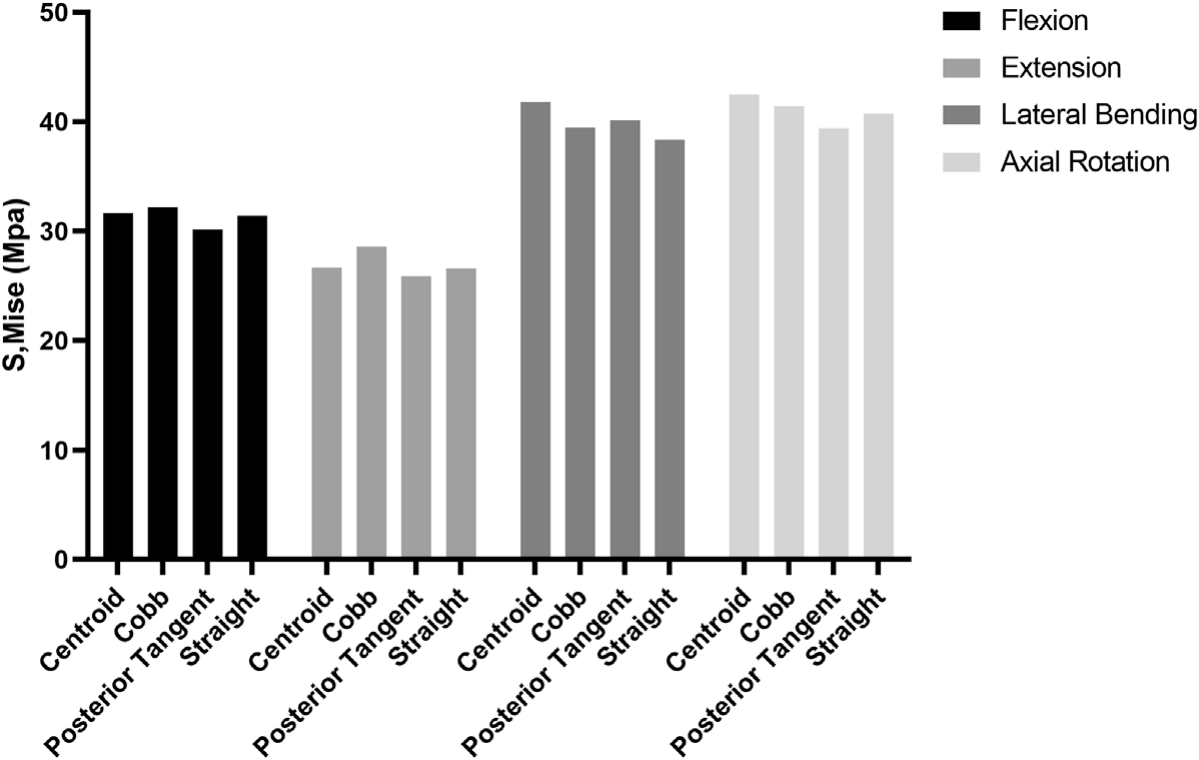
Comparison of maximum stress in fixation (MSF) for four RC models in flexion, extension, lateral bending, and axial rotation.

**FIGURE 7:**
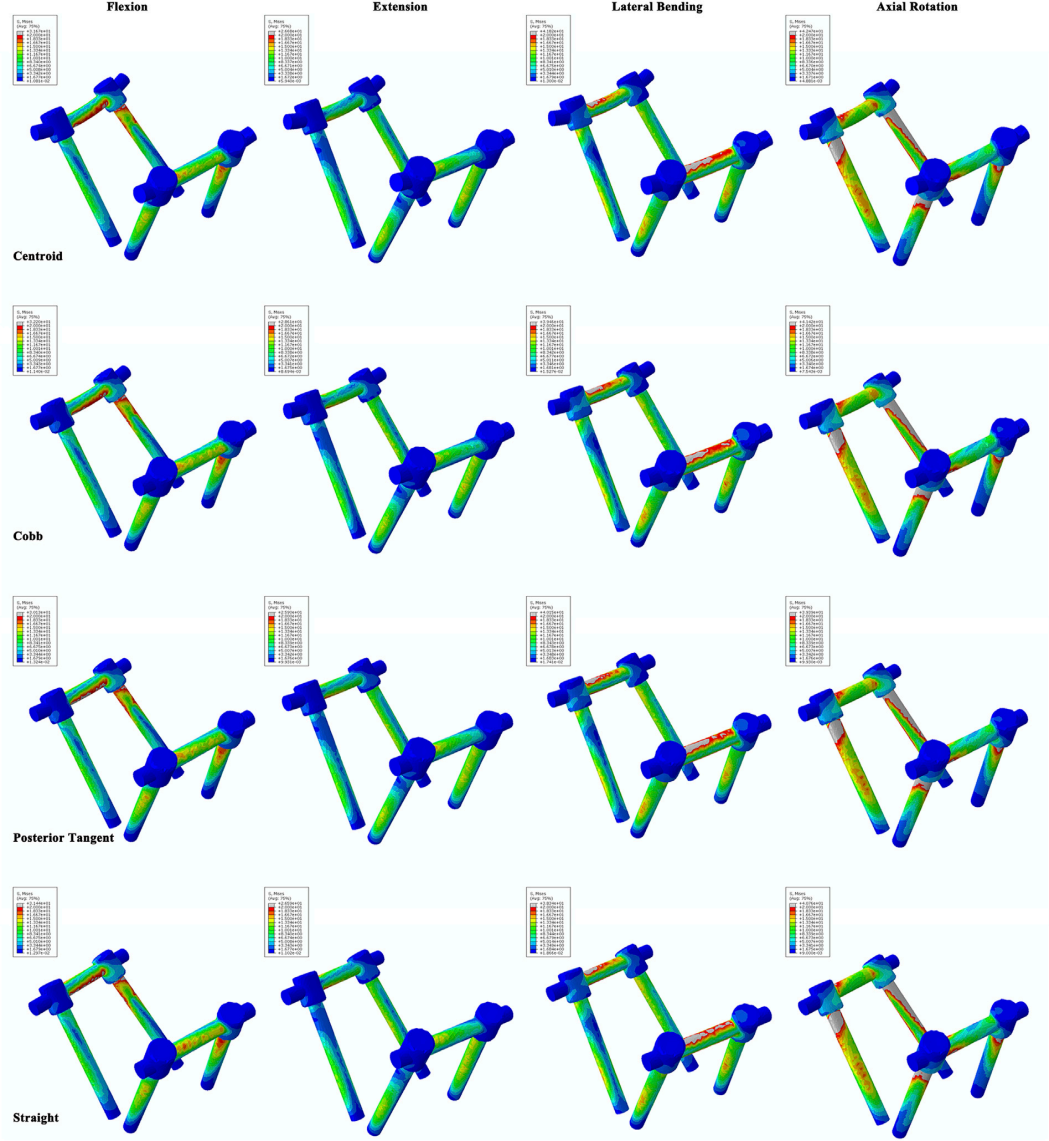
Stress contours of the screw–rod system for four RC models under flexion, extension, lateral bending, and axial rotation tests.

No difference in the ROM of the lumbosacral model in the single-segmental PLIF surgery was observed among four RC models under the four actions of flexion, extension, lateral bending, and axial rotation (Supplementary Figure S2 in Supplementary Materials). On the neighboring adjacent vertebrae (L3/4 and L5/S1) to the fused L4/5, there was also no substantial difference among four RC models in IRA and IDP with 7.5 N m moment under the four actions (Supplementary Figure S3 in Supplementary Materials). Stress distributions of the intervertebral discs and vertebrae showed a very weak difference between four RC models (Supplementary Figures S4, S5 in Supplementary Materials). The stress levels of collagen fibers were higher than those of annulus ground substance. In particular, MSFs under the four actions are illustrated in Figure 6. In flexion and extension, the Cobb model had the greatest stress, followed by the centroid model, straight model, and finally posterior tangent model. In the lateral bending and axial rotation, the greatest maximum stresses occurred in the centroid model; however, the posterior tangent model and Cobb model ranked the second and third, respectively, and the straight model was the last in the lateral bending, whereas in axial rotation, Cobb and straight models were in the middle, and posterior tangent had the minimum stress. The stress contours of the screw–rod system in four RC models with the values and locations of MSF under the four actions are shown in Figure 7. Overall, MSF in all RC models mainly occurred at the junction of screw and rods, and stress concentration was located at rods and thread run-out. In axial rotation, the screw–rod system experienced larger stress concentration than other actions.

### Clinical Results

**FIGURE 8:**
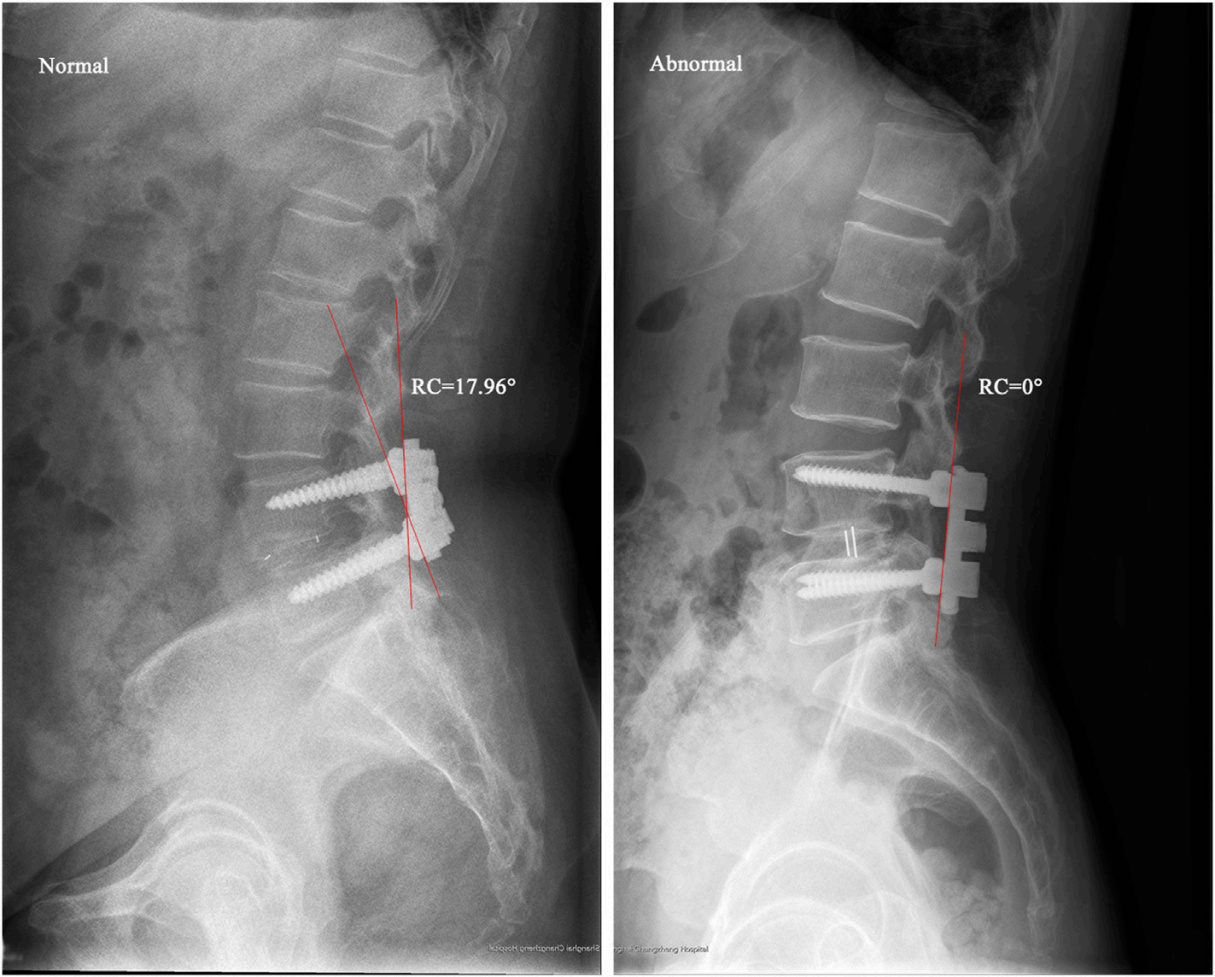
Typical cases showing rod contouring differences between normal and abnormal groups.

The analysis for characteristics of patients undergoing L4/5 segmental PLIF surgery were listed in Table 2. The primary diagnoses for reoperations were lumbar spinal stenosis at the adjacent level (10 cases) and internal fixation failure (2 cases). No significant differences were found between normal and abnormal groups in aspects of age, gender, BMI, surgical time, hospital stay, LL, PT, PI, SS, PI-LL, and PTA. The normal group had much higher RC (18.87° versus 4.70°, *p* < 0.01) and RC-PTA (6.18° versus −5.23°, *p* < 0.01) than the abnormal group (Figure 8).

**TABLE 2:** Patient demographics and radiographic parameters.

Variable	Normal (*N* = 19)	Revision (*N* = 12)	*p*-value
Age (years)	53.42 ± 10.05	52.71(0.15)	0.42
Gender (male/female)	10/9	9/3	0.27
BMI (kg/m^2^)	25.59 ± 3.32	23.49 ± 2.93	0.08
Surgical time (hour)	2.21 ± 0.38	2.46 ± 0.99	0.47
Hospital stay (days)	7.74 ± 3.33	6.67 ± 1.83	0.32
LL	52.62 ± 12.30	48.49 ± 7.97	0.31
PT	15.12 ± 6.95	18.79 ± 4.26	0.11
PI	51.12 ± 6.95	52.74 ± 8.67	0.67
SS	35.99 ± 7.52	33.95 ± 7.90	0.48
PI-LL	−1.51 ± 10.19	4.25 ± 7.84	0.11
RC	18.87 ± 6.16	4.70 ± 3.39	<0.01
PTA	12.68 ± 5.05	9.93 ± 3.09	0.10
RC-PTA	6.18 ± 7.96	−5.23 ± 4.71	<0.01

Abbreviation: LL, lumbar lordosis; PT, pelvic tilt; PI, pelvic incidence; SS, sacral slope; PI-LL, the difference between PI and LL; RC, rod curvature; PTA, posterior tangent angle of fused segments; RC-PTA, the difference between RC and PTA.

## Discussion

Optimization of spinal alignment measurements could provide guidance to the surgery. However, one question that needs to be answered is whether the rod contouring process should follow the same strategy. In the process of sagittal alignment correction, there is no evaluation on what RC could achieve a better clinical outcome. Thus, basing on three commonly-used LL measurements, we applied an analogy to rod contouring practice in spinal surgery and provided evidence for better rod contouring in single-segmental PLIF surgery by FE simulation. Although the three methods showed similar biomechanical properties under four actions, the posterior tangent method was relevantly superior in the fixation stress distributions of flexion, extension, and axial rotation. The RCs in centroid, Cobb, posterior tangent, and straight models were 33.25°, 18.65°, 7.99°, and 0°. Also, the RC-PTA of the four models were 25.26°, 10.66°, 0°, and −7.99°, respectively. The two variables of the posterior tangent model fell in the middle of the ranges. In the clinical study, it was demonstrated that moderate RC was beneficial for patients who underwent L4/5 segmental PLIF surgery, and negative RC-PTA was associated with adverse postoperative outcomes. Hence, both FEA and clinical study demonstrated that moderate rod contouring was necessary in L4/5 PLIF surgery. This result was partially consistent with Harrison’s finding with respect to algorithm comparison of LL assessment (Harrison et al., 2001). Combined with the analyzing methodologies, the posterior tangent method seemed better to fit normal spinal cord. Thus, we inferred that for the sake of convenience in clinical application, employing the fitting curve of the normal lumbar vertebral posterior edge or centerline of spinal canal to contour rod could be a feasible approach.

To date, improvement of surgical outcome and prevention of ASD-related complications are still the focus and challenge in lumbar surgery. In clinical practice, disappointing postoperative function and mechanical failure have been largely ascribed to inadequate restoration of sagittal alignment. Appropriate LL restoration was beneficial for reducing ASD risk. An *in vitro* biomechanical study reported that hypolordosis in the fused segments could reduce tension behavior of the anterior soft tissues and increase the stress of the posterior column of the adjacent vertebra (Umehara et al., 2000). Meanwhile, high stress of posterior internal fixation might increase risks of screw loosening or rupture. Moreover, decreased LL could increase the ROM and IDP of adjacent segments (Zhao et al., 2018), and excessive LL curvature also increased the IDP of the adjacent intervertebral disc (Wang et al., 2020). These also indicate the potential risk of ASD development. A meta-analysis revealed that post-operative LL was correlated with a significant increase in ASD occurrence after PLIF for degenerative lumbar disease (Wang and Ding, 2020). In this regard, to maintain proper physiologic lordosis at the fusion site, RC should be in accordance with the appropriate LL. This inference supplies the theoretical support for the current study to some extent. In addition, it must be pointed out that proper physiologic lordosis cannot be equated with originally normal lumbar alignment for degenerative lumbar disease. Vertebral osteophyte formation, intervertebral disc degeneration, facet hypertrophy, and compensatory change of surrounding tissues are the reflections of the body’s restabilization dealing with the lumbar pathological state in a long-term process. This spontaneous restabilization generates a special biomechanical status distinct from a patient’s normal lumbar condition or individuals with normal anatomic structure of lumbar spine. Thus, it requires proper correction of lumbar curvature based on clinical patient-specific conditions, and this challenges surgeons when evaluating spinal balance and planning correction of curvature.

To the best knowledge of the authors, there are few studies on the rod contouring optimization. Wanivenhaus et al. (2019) proposed that augmented reality-assisted rod bending could reduce operation duration by 20% compared with the traditional method and achieve higher accuracy of the rod bending process. Shi et al. (2020) confirmed that the Cobb angle could be used as reference to guide rod bending in thoracolumbar fractures and also reported that 4°–8° greater than the Cobb angle as the RC achieved the best spinal sagittal balance 2 years after the operation. Moufid et al. (2019) evaluated the mismatch between rod–screw and LL of the fused segment by the concept of the mismatch analysis index (MAI), which was determined by three parameters: the angle between the screw and rod, the angle between the superior endplate and screw, and the distance between the rod and posterior wall of vertebra. In addition, Wang et al. (2016) reported that increasing the bending angle of the concave rod, with respect to the convex rod, could improve the transverse plane correction with advantages in screw pullout forces and kyphosis.

The present study has a certain significance in the practical application of individualized or customized rod contouring before operation. Precise contouring could avoid the reduction of yield strength and stiffness caused by repetitive bending (Demura et al., 2015). It is worth noting that this study made a preliminary exploration on a habitual, pervasive, but subjective surgical procedure that requires standardization in the period of highly developing surgical techniques. Limitations should be acknowledged as well. First, this study did not take into account anatomical variations of the spinal structure. In fact, normal lumbar curvature varies greatly, which may result in different biomechanical performances of rod contouring. Spinal parameters, such as LL, PI, PT, and sacral slope (SS), are also associated with the sagittal balance. Second, the current study computationally modeled three commonly used lumbar lordosis measurements and straight rods to guide rod contouring. However, the modeled RCs should be solidified to have generalizability, interpretability, and clinical applicability by collecting more samples. Third, the clinical observations were patient-specific, and the real mechanical environment corresponding to the model boundary conditions varied, and this might lead to the discrepancy between the model and clinical observations. The patient-specific information should be further included to have exactly consistent results with the clinical observations. Fourth, FEA was a numerical evaluation of the biomechanical effect on the PLIF. The cadaveric study under cyclic loading and multicenter clinical studies with sound evidence should be conducted to verify the results. The accuracy of the radiographic parameter measurement could also be optimized by digital image processing and other analysis techniques. Nevertheless, there is no denying that the current study strongly controlled variables that inevitably occurred in clinical studies such as differences from patients and fixation placement.

## Conclusion

The present study studied the biomechanical effects of different RCs determined by centroid, Cobb, and posterior tangent methods on the single-segment PLIF surgery. The findings were that four RC models showed similar biomechanical behaviors under four actions, but the posterior tangent mode was relevantly superior to other three RC models in the aspect of fixation stress distribution. Moreover, the retrospective study further revealed that moderate RC and positive RC-PTA were associated with favorable prognosis.

## Data Availability

The original contributions presented in the study are included in the article/Supplementary Material, further inquiries can be directed to the corresponding authors.

## References

[B1] Ayturk U. M. Puttlitz C. M. (2011). Parametric Convergence Sensitivity and Validation of a Finite Element Model of the Human Lumbar Spine. Comput. Methods Biomech. Biomed. Eng. 14 (8), 695–705. 10.1080/10255842.2010.493517 21229413

[B2] Barrey C. Darnis A. (2015). Current Strategies for the Restoration of Adequate Lordosis during Lumbar Fusion. World J. Orthop. 6 (1), 117–126. 10.5312/wjo.v6.i1.117 25621216 PMC4303780

[B3] Chen Y.-L. (1999). Vertebral Centroid Measurement of Lumbar Lordosis Compared with the Cobb Technique. Spine (Phila Pa 1976) 24 (17), 1786–1790. 10.1097/00007632-199909010-00007 10488508

[B4] Demura S. Murakami H. Hayashi H. Kato S. Yoshioka K. Yokogawa N. (2015). Influence of Rod Contouring on Rod Strength and Stiffness in Spine Surgery. Orthopedics 38 (6), e520–523. 10.3928/01477447-20150603-61 26091226

[B5] Dreischarf M. Zander T. Shirazi-Adl A. Puttlitz C. M. Adam C. J. Chen C. S. (2014). Comparison of Eight Published Static Finite Element Models of the Intact Lumbar Spine: Predictive Power of Models Improves when Combined Together. J. Biomech. 47 (8), 1757–1766. 10.1016/j.jbiomech.2014.04.002 24767702

[B6] Glassman S. D. Berven S. Bridwell K. Horton W. Dimar J. R. (2005). Correlation of Radiographic Parameters and Clinical Symptoms in Adult Scoliosis. Spine (Phila Pa 1976) 30 (6), 682–688. 10.1097/01.brs.0000155425.04536.f7 15770185

[B28] Goel V. K. Park H. Kong W. (1994). Investigation of Vibration Characteristics of the Ligamentous Lumbar Spine Using the Finite Element Approach. J. Biomech. Eng. 116 (4), 377–383. 10.1115/1.2895787 7869712

[B7] Guo L.-X. Fan W. (2018). Dynamic Response of the Lumbar Spine to Whole-Body Vibration under a Compressive Follower Preload. Spine (Phila Pa 1976) 43 (3), E143–E153. 10.1097/BRS.0000000000002247 28538593

[B8] Harrison D. E. Harrison D. D. Cailliet R. Janik T. J. Holland B. (2001). Radiographic Analysis of Lumbar Lordosis: Centroid, Cobb, TRALL, and Harrison Posterior tangent Methods. Spine (Phila Pa 1976) 26 (11), E235–E242. 10.1097/00007632-200106010-00003 11389407

[B9] Huang H. Liu J. Wang L. Fan Y. (2021). A Critical Review on the Biomechanical Study of Cervical Interbody Fusion Cage. Med. Novel Technol. Devices 11, 100070. 10.1016/j.medntd.2021.100070

[B10] Janik T. J. Harrison D. D. Cailliet R. Troyanovich S. J. Harrison D. E. (1998). Can the Sagittal Lumbar Curvature Be Closely Approximated by an Ellipse? J. Orthop. Res. 16 (6), 766–770. 10.1002/jor.1100160620 9877403

[B11] Makhni M. C. Shillingford J. N. Laratta J. L. Hyun S.-J. Kim Y. J. (2018). Restoration of Sagittal Balance in Spinal Deformity Surgery. J. Korean Neurosurg. Soc. 61 (2), 167–179. 10.3340/jkns.2017.0404.013 29526059 PMC5853192

[B12] Matsumoto T. Okuda S. Maeno T. Yamashita T. Yamasaki R. Sugiura T. (2017). Spinopelvic Sagittal Imbalance as a Risk Factor for Adjacent-Segment Disease after Single-Segment Posterior Lumbar Interbody Fusion. J. Neurosurg. Spine 26 (4), 435–440. 10.3171/2016.9.SPINE16232 28059683

[B13] Moufid A. Y. Cloche T. Ghailane S. Ounajim A. Vendeuvre T. Gille O. (2019). Mismatch between Rod Bending and Actual Post-Operative Lordosis in Lumbar Arthrodesis with Poly Axial Screws. Orthop. Traumatol. Surg. Res. 105 (6), 1143–1148. 10.1016/j.otsr.2019.03.003 30928276

[B14] Naserkhaki S. Arjmand N. Shirazi-Adl A. Farahmand F. El-Rich M. (2018). Effects of Eight Different Ligament Property Datasets on Biomechanics of a Lumbar L4-L5 Finite Element Model. J. Biomech. 70, 33–42. 10.1016/j.jbiomech.2017.05.003 28549604

[B15] Patwardhan A. G. Havey R. M. Meade K. P. Lee B. Dunlap B. (1999). A Follower Load Increases the Load-Carrying Capacity of the Lumbar Spine in Compression. Spine (Phila Pa 1976) 24 (10), 1003–1009. 10.1097/00007632-199905150-00014 10332793

[B31] Polikeit A. Ferguson S. J. Nolte L. P. Orr T. E. (2003). Factors Influencing Stresses in the Lumbar Spine After the Insertion of Intervertebral Cages: Finite Element Analysis. Eur. Spine J. 12 (4), 413–420. 10.1007/s00586-002-0505-8 12955610 PMC3467788

[B16] Rohlmann A. Bauer L. Zander T. Bergmann G. Wilke H.-J. (2006). Determination of Trunk Muscle Forces for Flexion and Extension by Using a Validated Finite Element Model of the Lumbar Spine and Measured *In Vivo* Data. J. Biomech. 39 (6), 981–989. 10.1016/j.jbiomech.2005.02.019 16549091

[B17] Rohlmann A. Neller S. Claes L. Bergmann G. Wilke H.-J. (2001). Influence of a Follower Load on Intradiscal Pressure and Intersegmental Rotation of the Lumbar Spine. Spine (Phila Pa 1976) 26 (24), E557–E561. 10.1097/00007632-200112150-00014 11740371

[B18] Salmingo R. A. Tadano S. Abe Y. Ito M. (2014). Influence of Implant Rod Curvature on Sagittal Correction of Scoliosis Deformity. Spine J. 14 (8), 1432–1439. 10.1016/j.spinee.2013.08.042 24275616

[B30] Schmidt H. Heuer F. Drumm J. Klezl Z. Claes L. Wilke H. J. (2007). Application of a Calibration Method Provides More Realistic Results for a Finite Element Model of a Lumbar Spinal Segment. Clin Biomech. 22 (4), 377–384. 10.1016/j.clinbiomech.2006.11.008 17204355

[B19] Shi Z. Wang G. Jin Z. Wu T. Wang H. Sun J. (2020). Use of the Sagittal Cobb* Angle to Guide the Rod Bending in the Treatment of Thoracolumbar Fractures: A Retrospective Clinical Study. J. Orthop. Surg. Res. 15 (1), 574. 10.1186/s13018-020-02115-5 33256851 PMC7708173

[B29] Ueno K. Liu Y. K. (1987). A Three-Dimensional Nonlinear Finite Element Model of Lumbar Intervertebral Joint in Torsion. J. Biomech. Eng. 109 (3), 200–209. 10.1115/1.3138670 3657107

[B20] Umehara S. Zindrick M. R. Patwardhan A. G. Havey R. M. Vrbos L. A. Knight G. W. (20001976). The Biomechanical Effect of Postoperative Hypolordosis in Instrumented Lumbar Fusion on Instrumented and Adjacent Spinal Segments. Spine 25 (13), 1617–1624. 10.1097/00007632-200007010-00004 10870136

[B21] Wang K. Jiang C. Wang L. Wang H. Niu W. (2018). The Biomechanical Influence of Anterior Vertebral Body Osteophytes on the Lumbar Spine: A Finite Element Study. Spine J. 18 (12), 2288–2296. 10.1016/j.spinee.2018.07.001 29990595

[B22] Wang T. Ding W. (2020). Risk Factors for Adjacent Segment Degeneration after Posterior Lumbar Fusion Surgery in Treatment for Degenerative Lumbar Disorders: A Meta-Analysis. J. Orthop. Surg. Res. 15 (1), 582. 10.1186/s13018-020-02032-7 33272288 PMC7713357

[B23] Wang W. Pei B. Pei Y. Li H. Lu S. Wu X. (2020). Biomechanical Effects of over Lordotic Curvature after Spinal Fusion on Adjacent Intervertebral Discs under Continuous Compressive Load. Clin. Biomech. 73, 149–156. 10.1016/j.clinbiomech.2020.01.002 31986460

[B24] Wang X. Boyer L. Le Naveaux F. Schwend R. M. Aubin C.-E. (2016). How Does Differential Rod Contouring Contribute to 3-dimensional Correction and Affect the Bone-Screw Forces in Adolescent Idiopathic Scoliosis Instrumentation? Clin. Biomech. 39, 115–121. 10.1016/j.clinbiomech.2016.10.002 27750078

[B25] Wanivenhaus F. Neuhaus C. Liebmann F. Roner S. Spirig J. M. Farshad M. (2019). Augmented Reality-Assisted Rod Bending in Spinal Surgery. Spine J. 19 (10), 1687–1689. 10.1016/j.spinee.2019.06.019 31563336

[B26] Xia X.-P. Chen H.-L. Cheng H.-B. (2013). Prevalence of Adjacent Segment Degeneration after Spine Surgery. Spine 38 (7), 597–608. 10.1097/BRS.0b013e318273a2ea 22986837

[B27] Zhao X. Du L. Xie Y. Zhao J. (2018). Effect of Lumbar Lordosis on the Adjacent Segment in Transforaminal Lumbar Interbody Fusion: a Finite Element Analysis. World Neurosurg. 114, e114–e120. 10.1016/j.wneu.2018.02.073 29477002

