# Supplementary material for: Biomechanical and Clinical Study of Rod Curvature in Single-Segment Posterior Lumbar Interbody Fusion
Source: Front Bioeng Biotechnol. 2022 Mar 3;10:824688. doi: 10.3389/fbioe.2022.824688 (PMC8929399; doi:10.3389/fbioe.2022.824688)
Supplement: Supplementary file 1 [file DataSheet1.docx]

**Supplementary Materials**

**
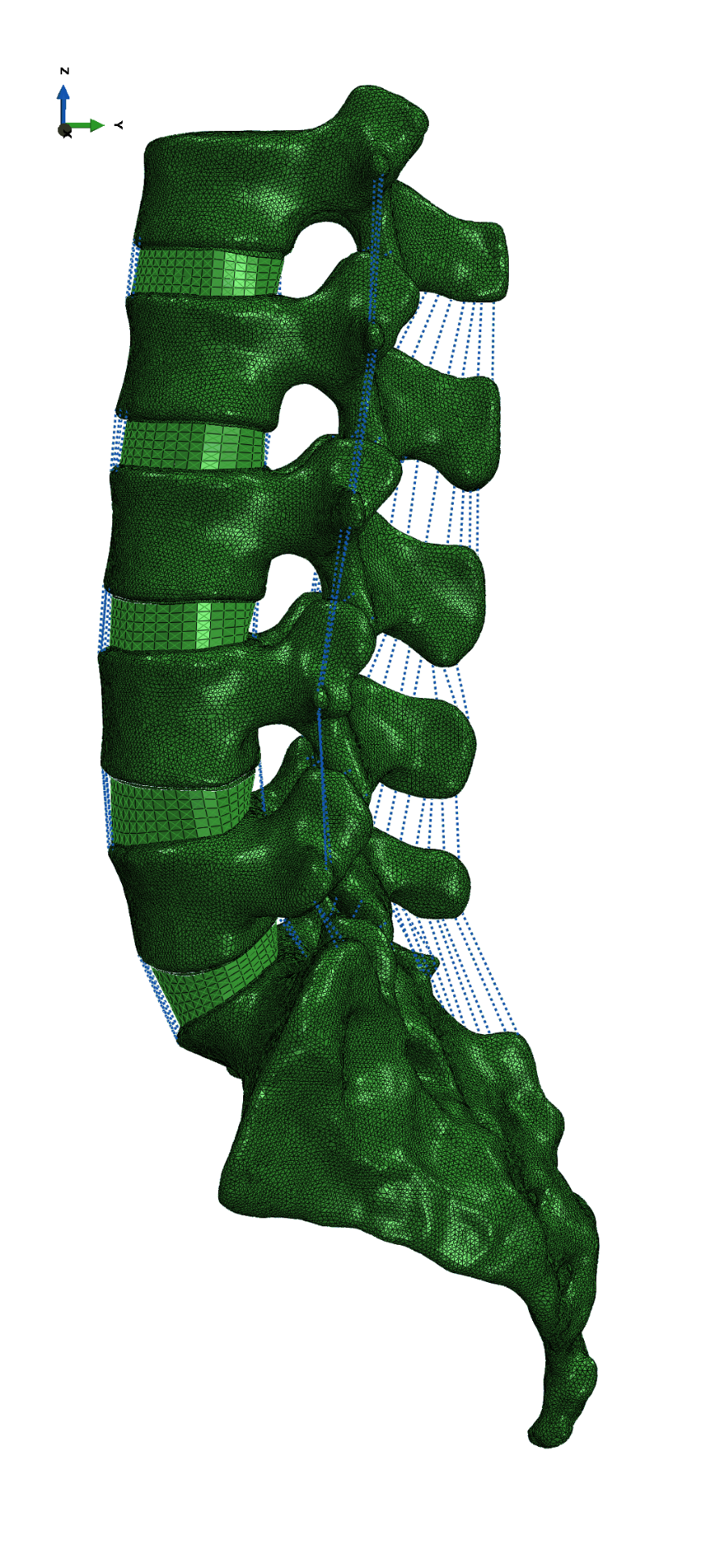
**

**Figure S1.** Normal lumbosacral model


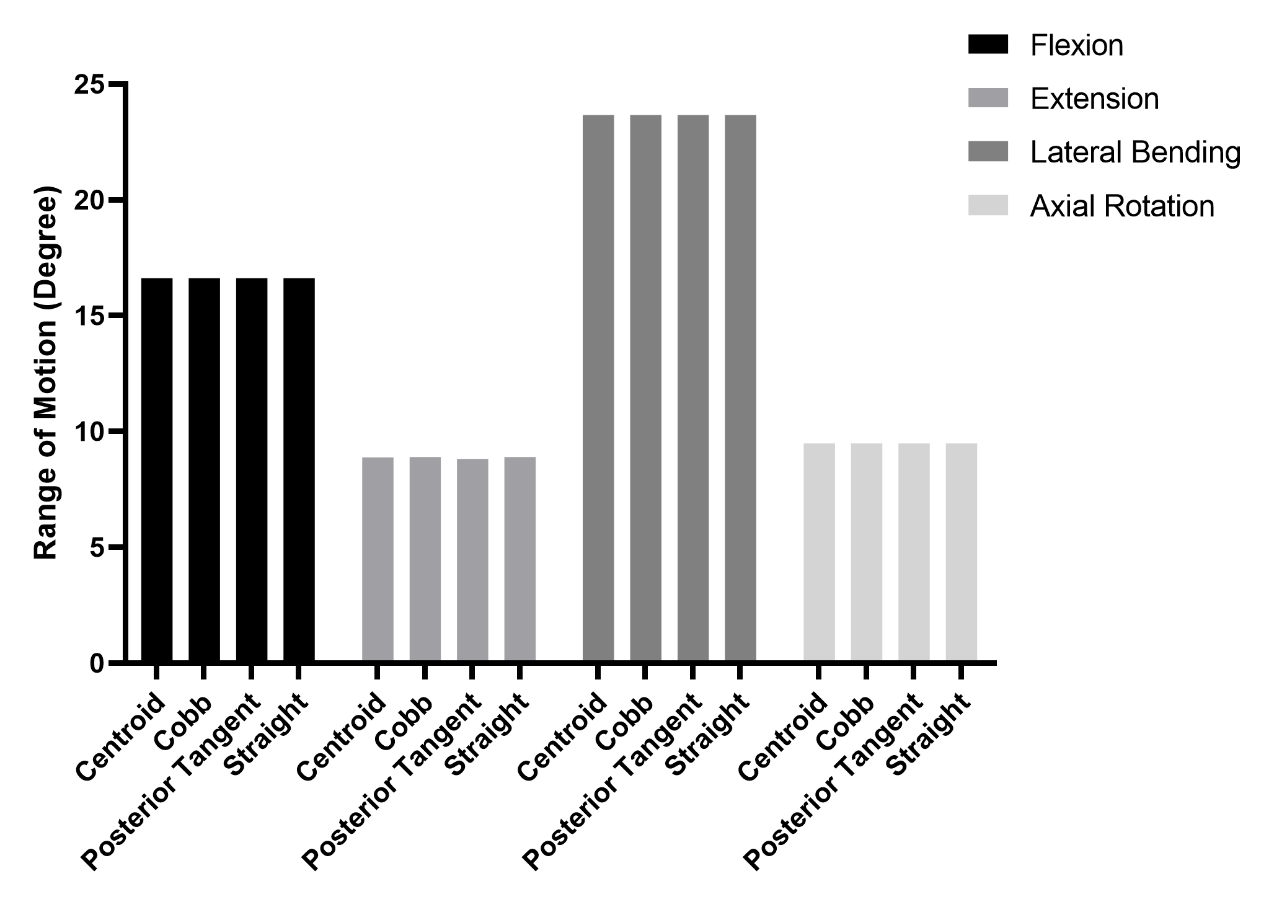


**Figure S2.** Comparison of Range of motion (ROM) for four models in flexion, extension, lateral bending and axial rotation.


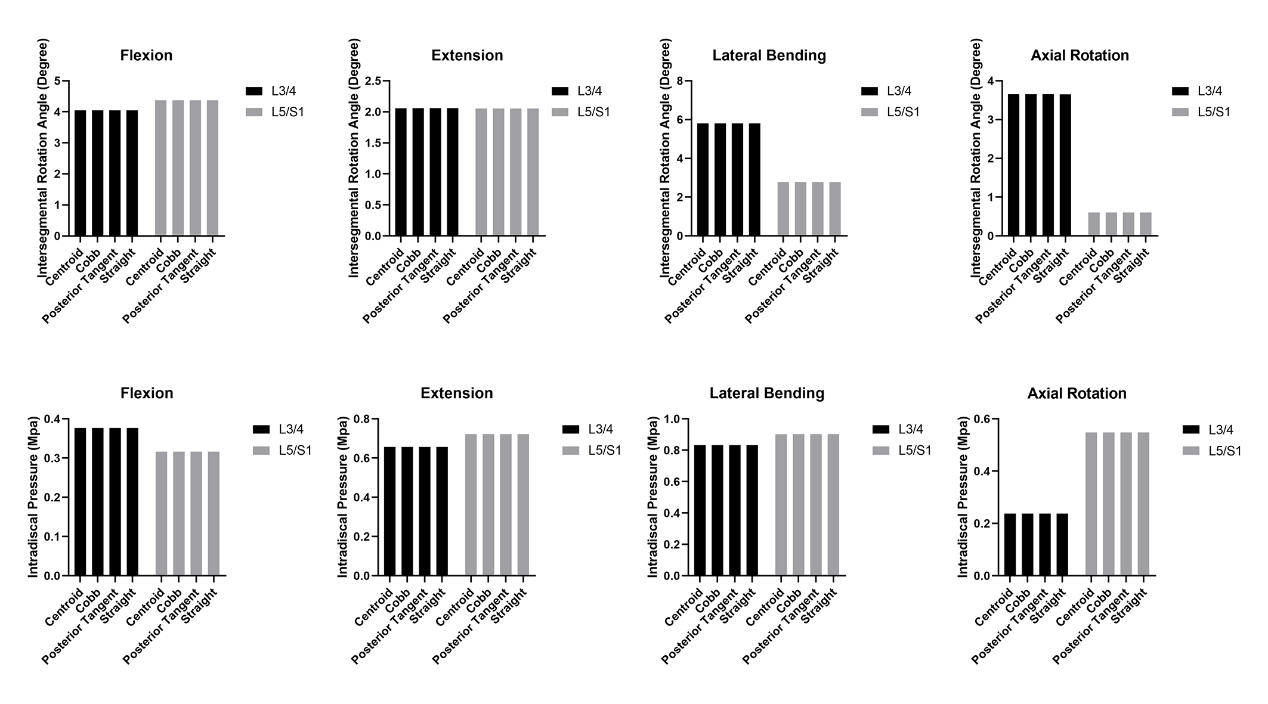


**Figure S3.** Comparison of intersegmental rotation angle (IRA) and intradiscal pressure (IDP) in adjacent segmental discs for four models in flexion, extension, lateral bending and axial rotation.


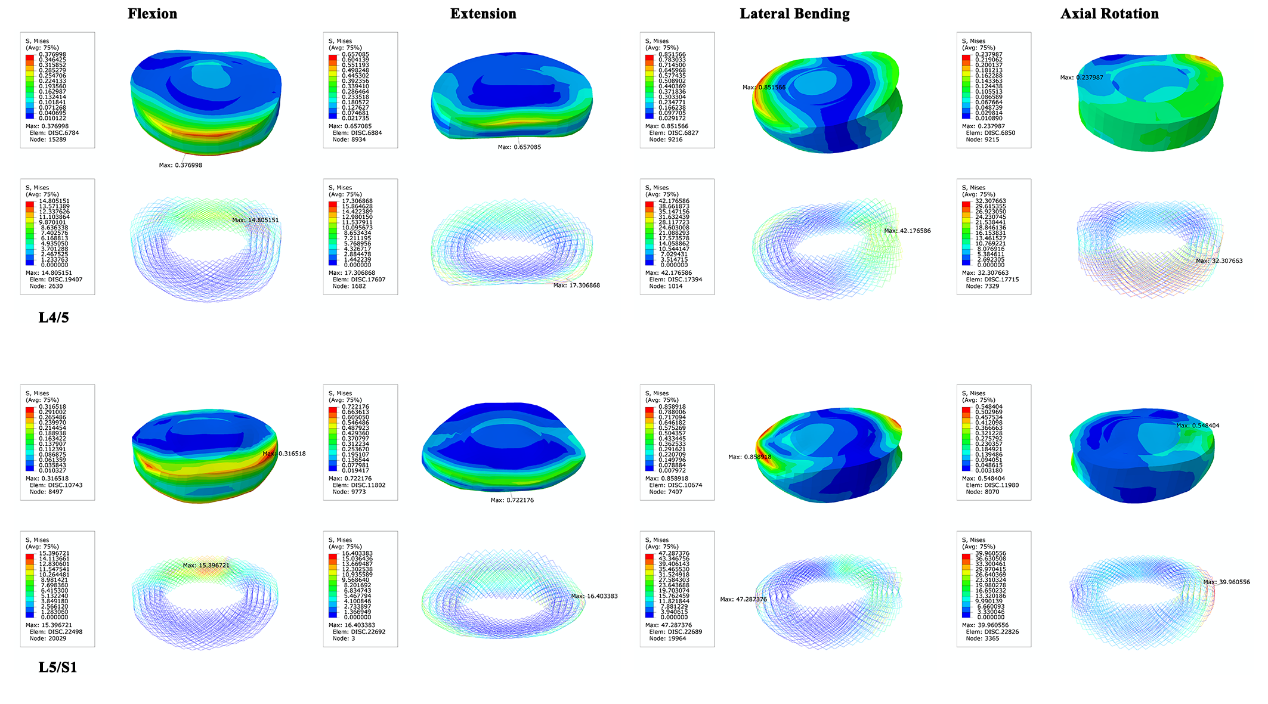


**Figure S4-1.** Stress contours of annular ground substance and nucleus pulposus, collagen fibers of intervertebral discs in centroid model.

**
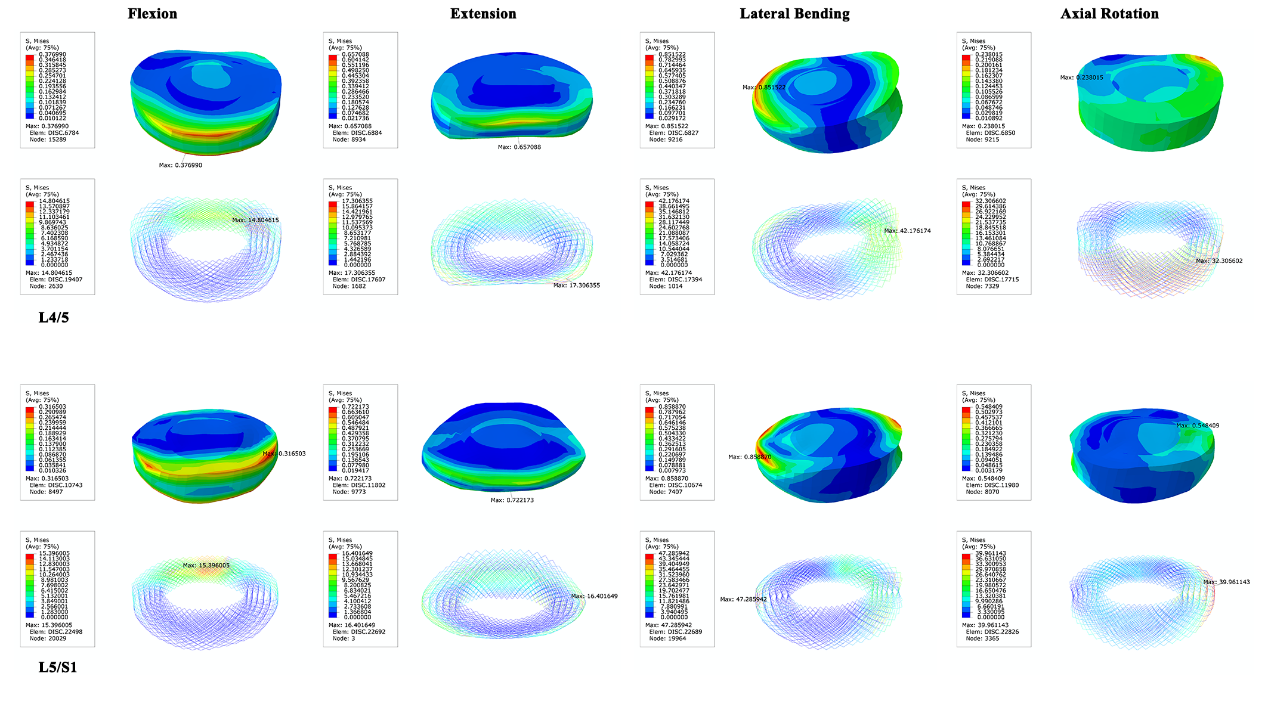
**

**Figure S4-2.** Stress contours of annular ground substance and nucleus pulposus, collagen fibers of intervertebral discs in Cobb model.


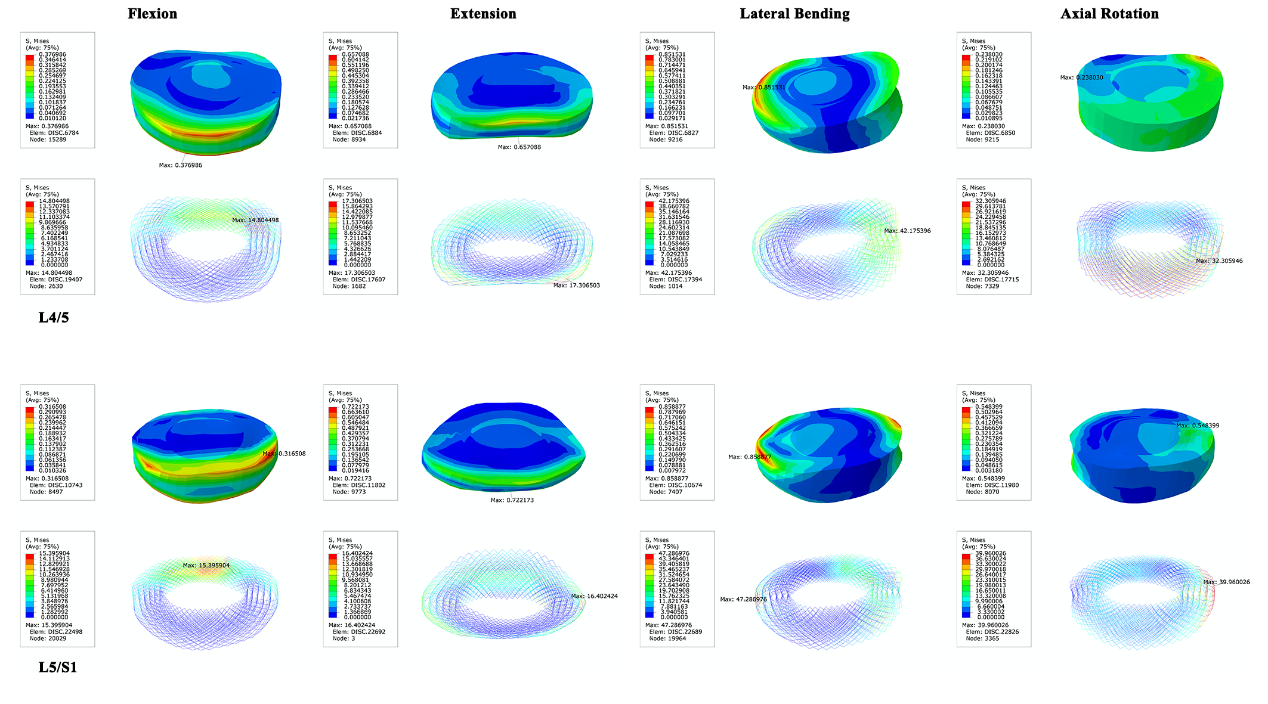


**Figure S4-3.** Stress contours of annular ground substance and nucleus pulposus, collagen fibers of intervertebral discs in posterior tangent model.


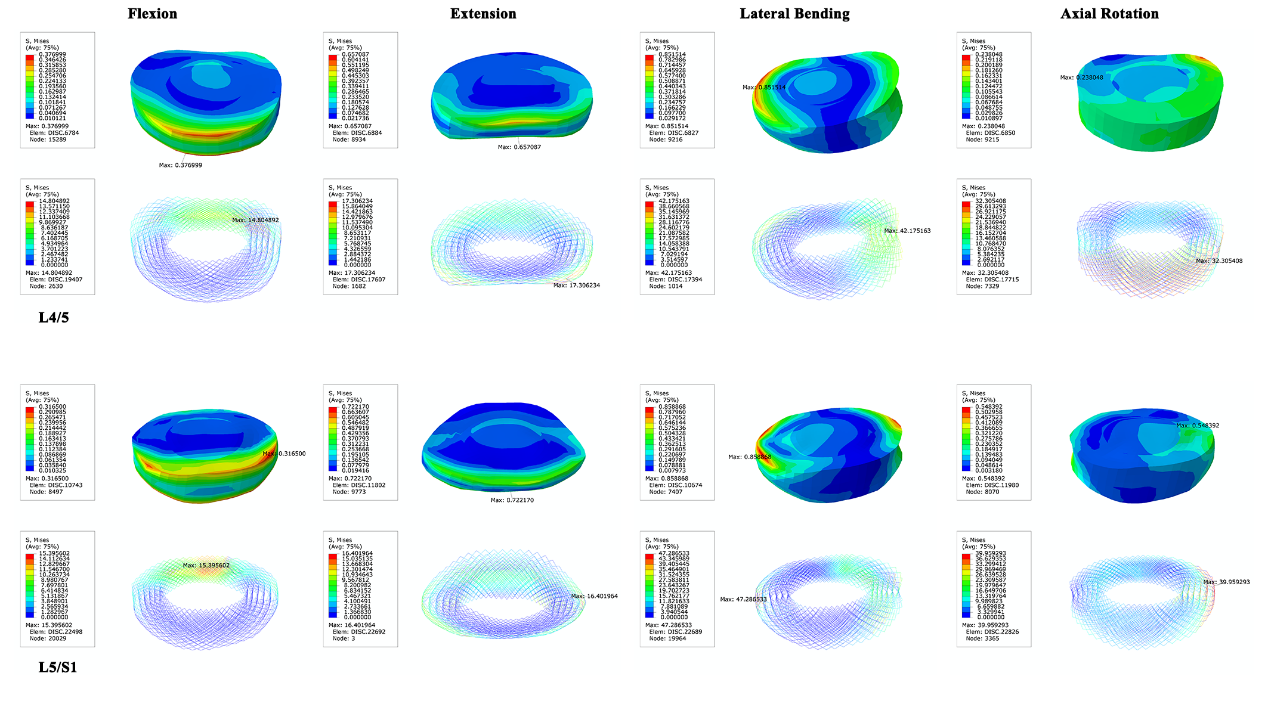


**Figure S4-4.** Stress contours of annular ground substance and nucleus pulposus, collagen fibers of intervertebral discs in straight model.

**
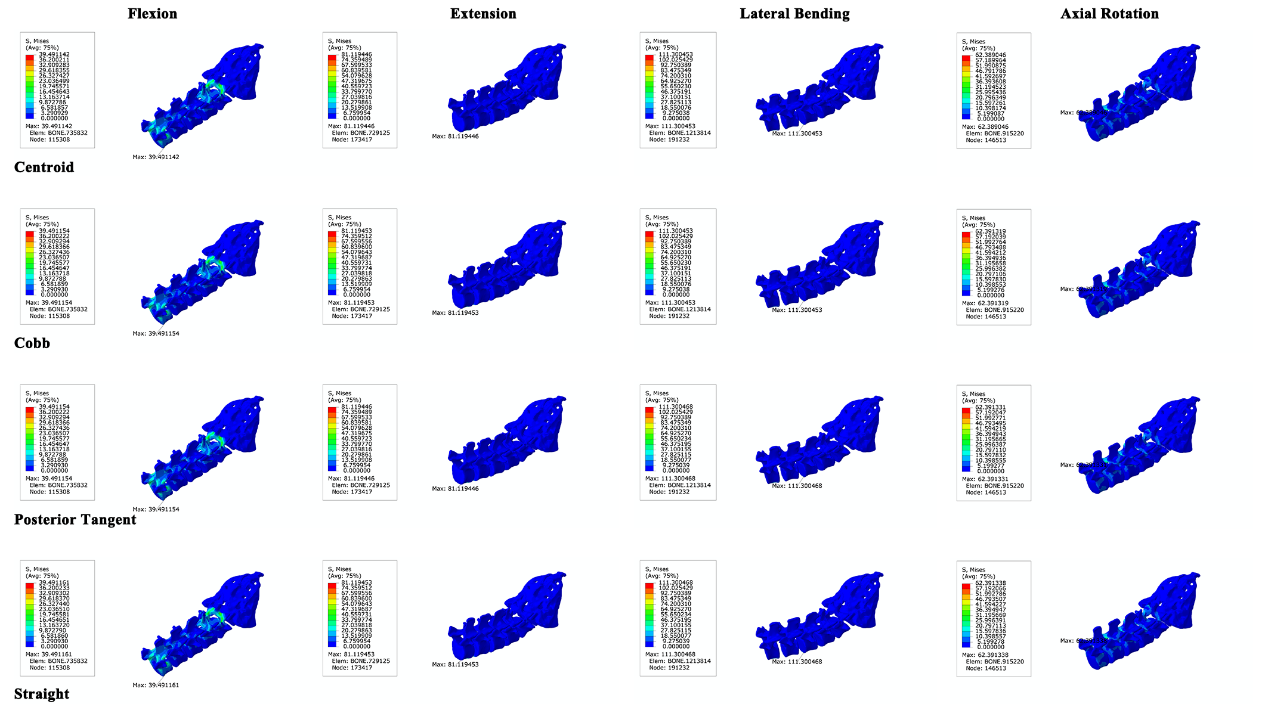
**

**Figure S5.** Stress contours of lumbosacral vertebrae.
